# Diabetic Neuropathy and Erectile Dysfunction: Unveiling the Neural Pathways Behind a Vascular Symptom

**DOI:** 10.3390/jcm15041621

**Published:** 2026-02-20

**Authors:** Virginia Zamponi, Rossella Mazzilli, Stefano Balducci, Antongiulio Faggiano, Jonida Haxhi

**Affiliations:** 1 Endocrinology Unit, Department of Clinical and Molecular Medicine, Sapienza University of Rome, Sant’ Andrea Hospital, 00189 Rome, Italy; rossella.mazzilli@uniroma1.it (R.M.); s.balducci@hctdiabete.it (S.B.); antongiulio.faggiano@uniroma1.it (A.F.); jonida.haxhi@uniroma1.it (J.H.); 2 Metabolic Fitness Association, 00015 Rome, Italy

**Keywords:** diabetes mellitus, erectile dysfunction, neuropathy, autonomic neuropathy, nitric oxide, BDNF, phosphodiesterase 5 inhibitors (PDE5-Is)

## Abstract

Erectile dysfunction (ED) is one of the most prevalent and disabling complications of diabetes mellitus (DM), thought to arise from the interaction of metabolic, vascular, and neural injury. Recent evidence indicates that diabetic neuropathy, affecting both somatic and autonomic pathways, plays a central role in the development of ED and is strongly associated with increased disease burden. Early neurophysiological studies documented impaired penile sensory conduction and abnormalities of sacral reflex pathways in diabetic men with ED, while more recent investigations have confirmed the contribution of cardiovascular autonomic neuropathy and small-fibre loss. At the molecular level, oxidative stress, advanced glycation end-product signalling, impaired nitric oxide bioavailability, and reduced neurotrophic support, particularly involving brain-derived neurotrophic factor (BDNF), emerge as key mechanisms linking diabetes to neural and neurovascular dysfunction. Although phosphodiesterase type-5 inhibitors remain first-line therapy, reduced responsiveness in patients with significant neuropathy highlights the importance of recognising the role of neurogenic mechanisms. Overall, the available evidence supports the conceptualisation of diabetic ED as a neurovascular manifestation within the broader spectrum of diabetic neuropathy rather than as a purely vasculogenic disorder. This review integrates historical and contemporary literature addressing the epidemiology, neurophysiology, pathophysiology and therapeutic implications of ED in diabetes, with a specific focus on its neuropathic substrate. These findings support a paradigm shift toward an integrated neurovascular approach to diabetic ED, highlighting the importance of early neuropathy-oriented assessment and paving the way for future regenerative and neuroprotective therapeutic strategies.

## 1. Introduction

Erectile dysfunction (ED) is a highly prevalent and disabling complication of diabetes mellitus (DM), affecting more than half of men over the course of the disease and exerting a significant impact on quality of life. A large systematic review and meta-analysis including both type 1 and type 2 DM reported a pooled prevalence of ED of 52.5%, with higher rates in type 2 DM and earlier onset in type 1 DM [1]. Although diabetic ED has historically been interpreted primarily as a vasculogenic condition, a growing body of evidence supports a pivotal role for diabetic neuropathy, including peripheral, autonomic, and nitrergic components, in its development and progression.

Several experimental, neurophysiological, and clinical studies have demonstrated that chronic hyperglycaemia, oxidative stress, and microvascular injury induce structural and functional alterations of penile innervation, leading to impaired neural signalling and defective neurovascular coupling [2,3,4,5]. These findings are reinforced by neurophysiological investigations showing abnormalities in dorsal penile nerve conduction, somatosensory evoked potentials, and sacral reflex pathways in individuals affected by DM with ED, often detectable even before overt sensory or autonomic symptoms elsewhere [6,7,8].

At the population level, large cohort studies and meta-analyses have further established a strong epidemiological association between diabetic neuropathy and ED. The presence of peripheral or autonomic neuropathy markedly increases the risk of ED independently of traditional cardiovascular risk factors, and in addition, ED may precede clinically apparent distal neuropathy or cardiovascular autonomic involvement [9,10]. These observations support the concept of ED as an early manifestation within the broader spectrum of diabetic neuropathy rather than as an isolated end-organ complication.

Despite this substantial body of evidence, the neurogenic dimension of diabetic ED remains relatively underrepresented in contemporary conceptual frameworks, which often emphasise vascular mechanisms and symptomatic treatment. As a result, the integration of neuropathic mechanisms into the interpretation, classification, and management of diabetic ED has been inconsistent across both research and clinical contexts.

The aim of this review is to provide an integrated overview of diabetic ED with a specific focus on its neuropathic substrate. By synthesising epidemiological data, pathophysiological mechanisms, neurophysiological evidence, and available therapeutic information, this review seeks to contextualise diabetic ED within the broader spectrum of diabetic neuropathy and to highlight its relevance as a harbinger of further neurovascular complications of DM.

## 2. Epidemiology of Diabetic Erectile Dysfunction

ED is one of the most prevalent and impactful complications of DM, and epidemiological evidence consistently shows that it occurs earlier, more frequently, and with greater severity in men with DM compared to those without DM. A systematic review and meta-analysis of 145 studies reported a pooled prevalence of 52.5% among men with DM, with higher rates of ED in type 2 DM and earlier onset in type 1 DM [1]. More recently, an umbrella review estimated that approximately two-thirds of diabetic men worldwide experience ED, with a pooled global prevalence of 65.8% (95% CI: 58.3–73.3%), confirming the substantial and widespread burden of ED in this population [11]. Additional high-quality national data come from the Italian Study Group on Erectile Dysfunction in Diabetes. In a prevalence study among nearly 10,000 diabetic men across 178 centres, ED was observed in 35.8%, showing strong associations with age, metabolic control, DM duration, and microvascular complications including neuropathy [12]. A companion analysis of type 1 and type 2 DM reported ED in 51% of men with type 1 DM but only 37% of those with type 2 DM, again highlighting the influence of glycaemic control, smoking, and BMI [13]. A further longitudinal study estimated an incidence rate of 68 cases per 1000 person-years over 2.8 years, with higher risk observed among older patients, those with type 2 DM, and patients with autonomic and sensory neuropathy [14].

Apparent differences in ED prevalence between type 1 and type 2 DM across studies likely reflect differences in age distribution, disease duration, metabolic profile, and assessment methods rather than true inconsistencies. Large population-based analyses and meta-analyses report higher overall ED prevalence in type 2 DM, largely driven by older age and a greater burden of metabolic and cardiovascular risk factors [1]. Conversely, studies adjusting for age and conducted in clinically characterised cohorts have shown higher ED prevalence in type 1 DM, likely reflecting longer disease duration, earlier exposure to chronic hyperglycaemia, and a greater contribution of diabetic neuropathy [12,13]. Variability in ED assessment methods further contributes to heterogeneity, as large epidemiological studies and meta-analyses often rely on validated patient-reported questionnaires such as the IIEF or SHIM, whereas earlier population-based and clinically characterised cohorts frequently used interview-based or non-standardised definitions, which may lead to lower prevalence estimates.

Diabetic neuropathy is similarly widespread, representing one of the most common microvascular complications of DM. A large systematic review estimated the global prevalence of diabetic peripheral neuropathy (DPN) at approximately 30–40% among individuals with DM [9], while the American Diabetes Association notes that both DPN and cardiovascular autonomic neuropathy (CAN) remain highly prevalent yet frequently underdiagnosed, particularly in long-standing DM [15].

The association between ED and neuropathy is robustly supported by clinical studies. In the Diabetes Control and Complications Trial/Epidemiology of Diabetes Interventions and Complications (DCCT/EDIC) study cohort, cardiovascular autonomic neuropathy (CAN) independently predicted the onset of ED and lower urinary tract symptoms over 17 years of follow-up in men with type 1 DM [10]. Clinical evidence shows that ED prevalence rises substantially in the presence of microvascular complications, including DPN and CAN [16]. An umbrella review identified diabetic neuropathy as one of the strongest predictors of ED in men with DM, with a pooled odds ratio of 3.27 (95% CI: 2.51–4.26), surpassing traditional cardiovascular risk factors [11].

Overall, epidemiological data demonstrate that ED and diabetic neuropathy are two highly prevalent and closely interconnected complications of DM. In fact, ED affects up to two-thirds of diabetic men, while neuropathy affects approximately one-third to one-half, and neuropathy consistently increases the risk of ED. These findings underscore the clinical relevance of assessing neuropathic involvement in diabetic men presenting with ED and support the consideration of ED as a clinical marker associated with underlying neural injury.

## 3. Pathophysiology of Diabetic Erectile Dysfunction

From a mechanistic standpoint, the pathophysiology of diabetic ED reflects a complex neurovascular disorder in which metabolic injury of peripheral and autonomic nerves interacts with microvascular damage and cavernosal smooth-muscle remodelling. Chronic hyperglycaemia, oxidative stress, and dyslipidaemia converge on penile nerves and endothelium, ultimately impairing nitric oxide (NO) signalling and neurovascular coupling. Experimental and clinical work indicate that neural injury often precedes, and then amplifies, vascular dysfunction, supporting the view of ED in DM as a neurovascular manifestation of systemic neuropathic disease rather than an exclusively vasculogenic condition [17].

### 3.1. Neurovascular Interplay and Biochemical Pathways

Hyperglycaemia is known to activate several interconnected biochemical cascades that jointly damage neural and endothelial structures. These include: (i) the polyol pathway, enhanced formation of advanced glycation end-products (AGEs); (ii) activation of protein kinase C (PKC) isoforms; (iii) increased production of reactive oxygen species (ROS); (iv) low-grade inflammation [18]. All of these components are crucial for unifying metabolic mechanisms described by Brownlee [18]. These processes have been shown to induce mitochondrial dysfunction and reduce nitric oxide synthase (NOS) expression and activity in both cavernous nerves and endothelium, thereby lowering NO bioavailability [18,19,20]. In parallel, hyperglycaemia and oxidative stress promote Rho-kinase–mediated enhancement of cavernosal smooth-muscle tone, a pathway implicated in the maintenance of penile flaccidity and vasoconstriction [21].

AGEs also promote cross-linking of extracellular matrix proteins and thickening of vascular and perineurial basement membranes, thereby impairing perfusion of the vasa nervorum. This mechanism is highlighted in the context of diabetic ED by Cellek et al. (2012) [17], who describe the contribution of microvascular insufficiency to nitrergic neuronal vulnerability. Histopathological and experimental models of diabetic neuropathy further demonstrate axonal degeneration, segmental demyelination, and Schwann-cell alterations consistent with chronic ischaemic–metabolic nerve injury [19].

These metabolic and microvascular changes ultimately reduce both neuronal (nNOS-derived) and endothelial (eNOS-derived) NO signalling, leading to impaired neurogenic and endothelium-dependent cavernosal relaxation [19,20]. Because NO released from cavernous nerves represents the initiating event for erection, early disruption of nitrergic fibres renders subsequent vascular damage clinically more relevant and contributes to reduced efficacy of phosphodiesterase 5 inhibitors (PDE5-Is) in advanced stages of diabetic ED [17].

Microvascular dysfunction of the vasa nervorum represents a critical pathophysiological bridge between neural and vascular injury in diabetic ED. Chronic hyperglycaemia induces endothelial dysfunction, basement membrane thickening, and reduced nitric oxide bioavailability within the microcirculation supplying peripheral and autonomic nerves, resulting in chronic endoneurial ischaemia and impaired nerve perfusion. Ischaemic injury of the vasa nervorum promotes axonal degeneration, demyelination, and impaired nerve regeneration, thereby amplifying neural vulnerability in DM [22], a mechanism consistent with contemporary frameworks describing diabetic neuropathy as a microvascular–metabolic disorder affecting sensory, autonomic, and nitrergic fibres [23]. In the context of erectile physiology, compromised perfusion of autonomic and nitrergic fibres innervating the corpora cavernosa disrupts neurovascular coupling, reinforcing the concept of diabetic ED as a neurovascular rather than purely vasculogenic disorder [24].

### 3.2. Neurotrophic and Regenerative Mechanisms

DM is thought to impair not only metabolic and vascular integrity but also the neurotrophic environment that supports autonomic and nitrergic neurons. Experimental and clinical evidence indicate that diabetes reduces the availability and signalling efficacy of brain-derived neurotrophic factor (BDNF), a key regulator of neuronal survival, axonal growth, and synaptic maintenance. In the context of erectile physiology, diminished signalling of brain-derived neurotrophic factor (BDNF)-tropomyosin receptor kinase B (TrkB) signalling compromises the resilience and regenerative potential of cavernous nerves, which are particularly vulnerable to oxidative stress and microvascular insufficiency.

Mechanistic studies show that BDNF potently stimulates neurite outgrowth in major pelvic ganglion (MPG) neurons and activates canonical survival pathways, including Janus kinase/signal transducer and activator of transcription (JAK/STAT), which enhance axonal regeneration and neuronal viability [25]. These pathways also support the functional recovery of nitrergic signalling, which is essential for cavernosal smooth-muscle relaxation. Experimental evidence further indicates that BDNF–TrkB signalling directly modulates neuronal nitric oxide synthase (nNOS) expression and activity in autonomic neurons. Reduced BDNF availability has been associated with impaired nitrergic neurotransmission and decreased nitric oxide release, thereby directly compromising neurogenic initiation of cavernosal relaxation in diabetic ED. Consistent with these findings, contemporary reviews report that deficits in neurotrophic signalling impair Schwann-cell function, slow axonal repair, and contribute to progressive nitrergic dysfunction in diabetic ED [26,27]

Collectively, these findings point to reduced neurotrophic support, persistent oxidative injury, and chronic microvascular ischaemia which create a state of neuroregenerative failure, in which damaged penile autonomic fibres exhibit limited capacity for recovery even when glycaemic control improves. This mechanistic framework highlights neurotrophic pathways—particularly BDNF–TrkB and JAK/STAT signalling—as potential targets for future regenerative approaches, although specific therapeutic agents have not yet been established for diabetic ED [25,27].

### 3.3. Neurophysiological and Neuroelectrographic Evidence

Neurophysiological investigations consistently demonstrate that both somatic and autonomic fibres contributing to penile innervation are affected in DM [3,4,28]. Early conduction studies showed a clear reduction in dorsal penile nerve conduction velocity in diabetic men with ED, often paralleling abnormalities in distal peripheral nerves and supporting the concept of a diffuse, length-dependent polyneuropathy [3,4,28]. In some cohorts, conduction slowing was detectable even before overt erectile symptoms, indicating that neurophysiology may reveal subclinical involvement.

Additional evidence comes from somatosensory evoked potentials, where prolonged cortical latencies and reduced amplitudes indicate impaired transmission along spinal and supraspinal sensory pathways [7,8]. Histological studies complement these findings, documenting reduced cholinergic, vasoactive intestinal peptide (VIP)-ergic, and adrenergic fibres in penile tissue of diabetic men with ED, consistent with widespread neurochemical denervation [29]. Electromyographic assessment of sacral reflex circuits further reinforces this pattern of neurochemical denervation. Abnormalities of the bulbocavernosus reflex, including prolonged latency and reduced amplitude, suggest dysfunction of pudendal afferent and efferent fibres at the S2–S4 level. Chronic neurogenic remodelling in perineal muscles with polyphasic reinnervation potentials has also been described in DM [5].

Quantitative sensory testing provides a broader physiological context. Diabetic men with ED display impaired thresholds for vibratory, thermal, and mechanical stimuli, reflecting dysfunction of Aβ, Aδ, and C fibres [2]. Large cohort studies have expanded this framework, showing that small-fibre pathology, assessed through intraepidermal nerve fibre density and corneal confocal microscopy, is strongly associated with ED severity in both type 1 and type 2 DM [30,31]. These abnormalities frequently occur despite preserved large-fibre conduction, highlighting the selective vulnerability of unmyelinated and thinly myelinated fibres.

Autonomic involvement is also well documented. Cardiovascular autonomic testing reveals reduced heart-rate variability and abnormal Valsalva responses among diabetic men with ED, pointing to a broader parasympathetic deficit [32]. Pupillometric studies similarly demonstrate reduced resting pupil diameter and impaired dilation dynamics, indicating systemic autonomic dysfunction that parallels alterations in genital autonomic pathways. In long-standing diabetes, autonomic neuropathy may extend beyond erectile impairment to include additional neuro-urological manifestations such as ejaculatory disorders, including retrograde ejaculation, further supporting the concept of a diffuse pelvic autonomic involvement in diabetes [33].

Taken together, neurographic, sensory, and autonomic findings converge on a model of mixed somatic–autonomic neuropathy, characterised by degeneration of myelinated and unmyelinated fibres, impaired central sensory conduction, and dysfunction of parasympathetic nitrergic pathways essential for cavernosal relaxation. This integrated neural impairment offers a coherent explanation for the severity of erectile dysfunction in longstanding diabetes and for the reduced responsiveness to PDE5-Is in patients with significant neuropathy.

**Figure 1:**
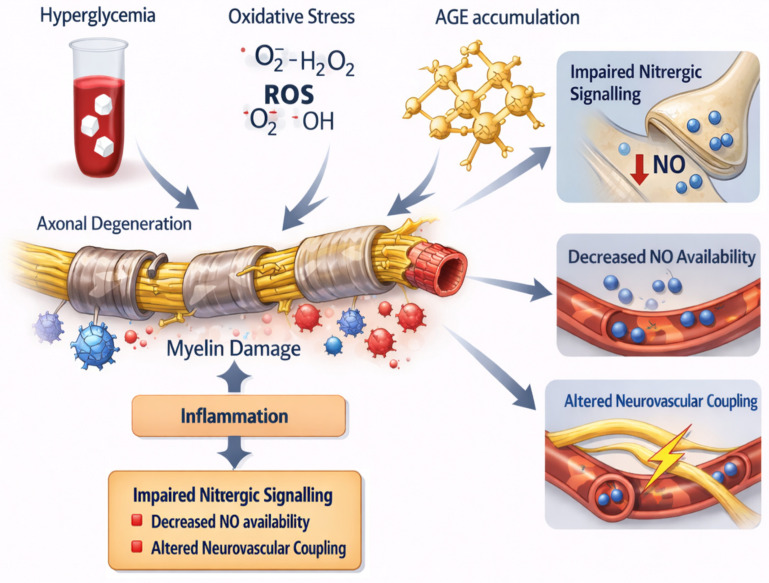
Neuropathic mechanisms underlying diabetic ED.

The main neuropathic mechanisms linking chronic hyperglycaemia to ED are summarised in Figure 1.

## 4. Clinical Manifestations and Assessment

The assessment of ED in men with DM should extend beyond a generic evaluation of sexual symptoms and explicitly consider ED as a potential manifestation of diabetic neuropathy. Clinical evaluation should focus on the temporal relationship between ED onset and DM duration, long-term glycaemic control, the presence of microvascular complications, neuropathic symptoms, and associated lower urinary tract complaints. In men with long-standing DM, ED frequently coexists with peripheral and autonomic neuropathy and may precede overt distal sensory deficits, supporting its role as an early marker of diffuse neural involvement rather than an isolated sexual disorder [10,31].

In this context, small-fibre dysfunction deserves particular attention, as it represents an early and often under-recognised component of diabetic neuropathy that may precede abnormalities detectable by conventional nerve conduction studies. Accordingly, emerging structural and imaging tools such as corneal confocal microscopy and intraepidermal nerve fibre density assessment through skin biopsy have gained increasing relevance for the objective evaluation of early neural involvement and may provide complementary support to clinical and questionnaire-based assessment. Both type 1 and type 2 diabetic cohorts show a strong association between reduced small-fibre density and ED severity, highlighting the contribution of autonomic and sensory small fibres to the erectile phenotype [30,31].

Quantitative sensory testing, together with corneal confocal microscopy, has emerged as a sensitive approach for detecting early involvement of unmyelinated and thinly myelinated fibres, frequently preceding abnormalities in conventional nerve conduction studies. Both type 1 and type 2 diabetic cohorts show a strong association between reduced small-fibre density and ED severity, highlighting the contribution of autonomic and sensory small fibres to the erectile phenotype [29,30].

Within this framework, validated questionnaires provide an essential quantitative anchor for symptom severity but do not fully capture the underlying pathophysiological substrate. The International Index of Erectile Function (IIEF-15 or IIEF-5) remains the most extensively validated instrument for multidimensional assessment of erectile function and is widely used in both clinical trials and routine practice [34]. Scores derived from the erectile function domain allow severity stratification and longitudinal monitoring; however, they do not discriminate between vasculogenic and neurogenic mechanisms, a limitation of particular relevance in diabetic populations, in whom erectile symptoms may precede clinically overt neuropathy and questionnaire scores may underestimate early or predominantly neurogenic dysfunction.

For this reason, in men with DM, especially when neuropathic involvement is clinically plausible, questionnaire-based assessment should be interpreted alongside structured screening for peripheral and autonomic neuropathy. Simple bedside tools such as the Michigan Neuropathy Screening Instrument or comparable clinical scores are recommended for the evaluation of distal symmetric polyneuropathy and are endorsed by international DM guidelines [15,35]. Evidence from cohort studies indicates that ED often reflects a broader neuropathic burden rather than an isolated sexual complaint, reinforcing the need for integrated neurological assessment [31].

Assessment of autonomic dysfunction further refines diagnostic classification. Cardiovascular autonomic reflex tests, including heart-rate variability analysis, the expiration-to-inspiration (E:I) ratio, and Valsalva manoeuvres, provide objective evidence of parasympathetic impairment that often parallels erectile symptoms. The American Diabetes Association explicitly recognises ED as a manifestation of diabetic autonomic neuropathy and recommends active screening for autonomic complications, including sexual dysfunction, particularly in individuals with long-standing DM or other microvascular involvement [15,35].

Neurophysiological investigations, such as dorsal penile nerve somatosensory evoked potentials and bulbocavernosus reflex latency, are not required in routine clinical practice but retain value in selected cases. These include younger patients with long-standing DM and minimal macrovascular disease, men with ED refractory to PDE5-Is, and individuals with atypical or discordant clinical features. In such contexts, neurophysiological testing can substantiate a predominantly neurogenic mechanism, as demonstrated by studies documenting delayed conduction, prolonged cortical latencies, and abnormal sacral reflexes in diabetic men with ED [5,6,7].

Laboratory evaluation complements the diagnostic work-up and should include assessment of glycaemic control, lipid profile, renal function, and androgen status. Hypogonadism, which is common in men with type 2 DM, may exacerbate endothelial and neural dysfunction and contribute to treatment resistance, although it does not fully explain neuropathic erectile impairment [16].

Penile duplex Doppler ultrasound remains a useful second-line tool when differentiation between vasculogenic and neurogenic components is clinically necessary. However, in diabetic men, normal penile haemodynamics do not exclude a neuropathic origin of ED. Recent position papers emphasise that Doppler findings should be interpreted within an integrated clinical and neuroendocrine framework, particularly in patients with DM, where neurogenic mechanisms frequently coexist with or precede vascular abnormalities [36].

Overall, assessment of diabetic ED should follow an integrated and pathophysiology-oriented approach, combining symptom quantification, neuropathy screening, autonomic evaluation, and targeted investigations. This strategy allows ED to be correctly interpreted as a manifestation of systemic neuropathy, facilitating accurate phenotyping, prognostic stratification, and personalised therapeutic planning.

At present, no validated, stepwise assessment pathway specifically tailored to diabetic ED exists, and clinical evaluation is typically guided by patient characteristics, disease stage, and available resources. Future research should therefore focus on the development of integrated diagnostic frameworks combining clinical history, metabolic and hormonal assessment, symptom-based questionnaires, and targeted evaluation of peripheral and autonomic neuropathy to improve phenotyping and clinical decision-making.

The principal neuropathic domains involved in DED, together with the corresponding assessment tools and clinical implications, are summarised in Table 1.

## 5. Management and Therapeutic Approaches

The management of ED in men with DM should be framed within a multimodal and pathophysiology-oriented strategy that addresses both the underlying metabolic–neuropathic substrate and the need for effective symptomatic relief. In this context, diabetic ED should not be regarded solely as a local vascular disorder but as a clinical manifestation of systemic neurovascular injury, in which metabolic control, neuropathic burden, and endothelial dysfunction jointly influence disease progression and treatment response.

Accordingly, therapeutic strategies should aim not only to restore erectile function but also to mitigate the progression of diabetic neuropathy and microvascular damage, which represent key determinants of long-term outcomes and responsiveness to pro-erectile therapies.

### 5.1. Antidiabetics Therapies and ED

Optimisation of glycaemic control remains the cornerstone of prevention and long-term management of diabetic ED. Longitudinal evidence from the DCCT/EDIC study demonstrates that intensive glycaemic control significantly reduces the incidence and progression of diabetic neuropathy and other microvascular complications, which in turn are strongly associated with the subsequent development of erectile dysfunction and lower urinary tract symptoms [10]. Current international recommendations emphasise the importance of improving time-in-range and reducing glycaemic variability, as these parameters are increasingly recognised to influence neurological outcomes and the long-term burden of diabetes-related complications, including those affecting sexual health [33,35]. Alongside glycaemic optimisation, aggressive management of cardiovascular risk factors, including hypertension, dyslipidaemia, obesity, and smoking cessation, is universally recommended, given their contribution to oxidative stress, microvascular ischaemia, and neural injury [34,36].

Beyond global metabolic control, the potential impact of specific glucose-lowering agents on erectile function has gained increasing attention. Although most antidiabetic therapies have not been specifically evaluated for neurogenic ED endpoints, available data suggest heterogeneous effects on vascular, metabolic, and possibly neural pathways relevant to erectile physiology. Metformin remains the first-line pharmacological therapy in type 2 DM and may exert indirect benefits on erectile function through improvements in insulin sensitivity, endothelial function, and systemic inflammation, although direct evidence on neurogenic mechanisms remains limited. Similarly, insulin therapy and traditional oral agents such as sulfonylureas primarily influence erectile outcomes through improved glycaemic control rather than through specific neurovascular effects [37].

Sodium–glucose cotransporter-2 inhibitors (SGLT2i) have shown favourable cardiovascular and metabolic effects that may indirectly support erectile function, including weight reduction, improved endothelial function, and attenuation of systemic inflammation. Preliminary clinical observations suggest potential improvements in sexual function in men with type 2 DM treated with SGLT2i, although robust data specifically addressing neuropathic erectile dysfunction are still lacking and causality remains uncertain. Their role in this context therefore appears supportive rather than directly disease-modifying [37].

Emerging evidence suggests that glucagon-like peptide-1 receptor agonists (GLP-1RAs) may exert indirect benefits on erectile function in men with type 2 diabetes [37]. Retrospective cohorts indicate that GLP-1RAs added to metformin are associated with improved erectile performance compared with metformin alone [38], and exploratory analyses of the placebo-controlled REWIND trial suggest a modest reduction in the incidence of moderate to severe erectile dysfunction with dulaglutide [39]. Systematic analyses further report a favourable effect of GLP-1RAs on erectile function compared with conventional therapies, particularly in individuals with obesity [40]. Mechanistically, these associations likely reflect improvements in endothelial function, weight reduction, and overall metabolic health rather than direct modulation of neurogenic pathways, and causality remains to be established in randomised trials designed with sexual/neurogenic endpoints [41].

### 5.2. Pro-Erectile Therapies and Diabetic ED

PDE5-Is remain the first-line pharmacological therapy for erectile dysfunction according to European and Italian National guidelines; however, their effectiveness is consistently reduced in men with DM and established peripheral or autonomic neuropathy [36,42]. In this specific subgroup, response rates are substantially lower than those reported in unselected diabetic populations and frequently fall below 50%, reflecting the reliance of PDE5-Is on preserved nitrergic signalling and intact neurovascular coupling [43,44]. It is likely that neuropathic involvement could represent a major determinant of therapeutic failure, as diabetic polyneuropathy and cardiovascular autonomic neuropathy impair nitric oxide bioavailability, endothelial responsiveness, and neural transmission within the erectile pathway. Consistently, both randomised trials and real-world data indicate that men with more advanced neuropathic burden, longer DM duration, and poorer glycometabolic control are more likely to be partial or complete non-responders to oral therapy [43,44]. Experimental and translational evidence further suggests that PDE5-Is may exert favourable effects on neurovascular perfusion and microcirculatory function, particularly when administered at early stages of diabetic neuropathy; however, available human data do not support a true disease-modifying effect on established neuropathic damage, and their clinical benefit in diabetic ED remains predominantly symptomatic [45]. Accordingly, PDE5-Is should not be expected to reverse the underlying neuropathic process but rather to provide functional improvement when sufficient neural integrity is preserved.

In clinical practice, therefore, the management of ED in men with diabetic neuropathy should adopt a pathophysiology-oriented approach, initiating PDE5-Is early alongside strict glycometabolic optimisation, aggressive cardiovascular risk management, and regular reassessment of their effectiveness. In patients with established neuropathy and inadequate response to oral therapies, alternative or combination strategies, including intracavernosal prostaglandin E1 or device-based treatments, should be promptly considered, as outlined in current guidelines [36,42]. Within this framework, therapeutic escalation should be guided by the extent of neuropathic involvement and by the persistence of ED despite optimised first-line management.

When first-line therapy fails or is contraindicated, second-line erectile treatments should be considered such as with intracavernosal prostaglandin E1 which represents the most established and effective option in diabetic ED [46]. Both the European Association of Urology and SIAMS guidelines identify intracavernosal alprostadil as the treatment of choice for patients who do not respond to PDE5-Is, including those with advanced diabetic neuropathy [36,42]. By directly inducing cavernosal smooth-muscle relaxation via cyclic AMP–dependent pathways, prostaglandins bypass the need for intact neural initiation of erection and therefore retain efficacy in clinical settings characterised by autonomic and nitrergic dysfunction, although they do not address the underlying neuropathic process. Clinical series and guideline-based recommendations consistently report preserved response rates to intracavernosal therapy in diabetic men with severe neuropathic involvement, supporting its role as a key therapeutic option when oral treatments fail [36,42]. Accordingly, intracavernosal prostaglandin therapy represents a pathophysiologically sound and guideline-endorsed strategy for restoring erectile function in men with neuropathic diabetic ED.

### 5.3. Adjunctive Therapies

Adjunctive therapies targeting diabetic neuropathy may complement standard erectile treatments, and although clinical evidence on erectile outcomes remains limited, small controlled studies suggest potential additive benefits when neuropathy-directed therapies are combined with other erectile treatments. Vacuum erection devices (VEDs) and structured sexual rehabilitation should be considered adjunctive options in men with diabetic ED with suspected or established neuropathic involvement. By inducing tumescence mechanically, VEDs bypass impaired autonomic and nitrergic signalling and can provide functional erections, although real-world persistence is variable; guideline summaries report high initial efficacy with a decline over time, supporting their use mainly as a supportive strategy in selected patients and complex cases [36,42]. Meta-analytic data suggest that VED efficacy remains clinically relevant also in DM, with pooled response rates around 70%, and that combination approaches may enhance outcomes in difficult-to-treat phenotypes [47]. In diabetic men with suboptimal response to oral therapy, adding VED to PDE5-Is has been shown to improve erectile outcomes compared with pharmacological therapy alone, supporting an “add-on” strategy when neural impairment limits oral efficacy [48,49].

Low-intensity extracorporeal shockwave therapy (Li-ESWT) has emerged as a potential regenerative-oriented modality; however, evidence in diabetic ED remains heterogeneous. Clinical studies in diabetic cohorts, including PDE5-Is non-responders and patients with polyneuropathy, have reported variable improvements in erectile function, often within multimodal protocols or combination strategies, but with inconsistent durability and magnitude of benefit [50,51,52]. Reviews and pooled analyses indicate that Li-ESWT may convert a proportion of diabetic non-responders into pharmacological responders, yet substantial heterogeneity in treatment protocols and outcome measures limits firm conclusions [53,54]. Accordingly, guidelines consider Li-ESWT an optional or investigational therapy in diabetes-associated ED, emphasising the lack of standardisation and the limited evidence in patients with advanced neuropathic involvement [36,42,55].

In patients with early or moderate neural involvement, alpha-lipoic acid and related neurotrophic agents have shown modest but consistent benefits on symptoms and electrophysiological measures of diabetic peripheral neuropathy [56]. Given the established pathophysiological link between oxidative stress, microvascular dysfunction, and autonomic nerve impairment in diabetic erectile dysfunction, these agents may support neural function within erectile pathways [10,57].

Improvements in erectile function scores are seen with alprostadil plus alpha-lipoic acid or nutraceutical combinations in selected diabetic cohorts [58,59]. However, these interventions should be regarded as complementary rather than disease-modifying in neuropathic diabetic ED.

Future therapeutic strategies for diabetic ED with a neuropathic component are increasingly focused on interventions aimed at restoring neural integrity rather than providing purely symptomatic relief. Experimental evidence indicates that neurotrophic and neuromodulatory approaches can promote cavernous nerve preservation and regeneration, thereby addressing a key pathophysiological substrate of neurogenic ED. In animal models, neurotrophins such as BDNF have been shown to enhance neurite outgrowth, preserve nitrergic signalling, and improve erectile responses following cavernous nerve injury or diabetic insult, primarily through activation of TrkB-dependent pathways and downstream JAK/STAT signalling [60,61]. Translation to human disease, however, remains challenging. The only randomised, double-blind clinical trial evaluating recombinant human BDNF in diabetic polyneuropathy failed to demonstrate significant improvements in conventional nerve conduction or sensory endpoints, although post hoc analyses suggested potential benefits in selected small-fibre domains, highlighting the importance of patient selection and outcome measures in future studies [62].

Stem cell–based approaches and their paracrine derivatives represent another promising avenue. Preclinical studies consistently show that adipose-derived stem cells and, more recently, stem cell–derived exosomes can improve erectile function in diabetic animal models by reducing apoptosis of cavernosal endothelial and smooth muscle cells, enhancing endothelial markers, and partially restoring neurovascular homeostasis [63]. Despite these encouraging data, current evidence remains largely confined to experimental models, and no disease-modifying therapy targeting diabetic neuropathy has yet demonstrated robust efficacy in restoring erectile function in humans.

## 6. Conclusions

Diabetic ED emerges as a clinically accessible window into the broader landscape of diabetic neuropathy. Far from being an isolated sexual disorder, ED reflects the vulnerability of autonomic and somatic neural networks to chronic metabolic stress, positioning penile function among the earliest and most sensitive indicators of diffuse neural injury in DM. This perspective challenges the traditional vascular-centric view of ED and reframes ED as a neurovascular endpoint of systemic neuropathic disease.

Diabetic ED may be read as a quiet signal of neural fragility, emerging from the earliest disruption of autonomic and nitrergic pathways, often preceding overt sensory loss or structural vascular disease. In this light, sexual dysfunction in DM can be considered a functional expression of neuropathic burden, challenging regenerative therapies to elevate sexual health to the same disease-modifying horizon as DM itself.

From a clinical and translational perspective, this view underscores the unmet need for neurotrophic or neuroregenerative therapies and for the identification of reliable biomarkers of neurogenic ED to enable earlier recognition, patient stratification, and targeted intervention.

Chronic hyperglycaemia promotes oxidative stress through reactive oxygen species (ROS) and leads to the formation and accumulation of advanced glycation end-products (AGEs). These metabolic insults induce axonal degeneration, myelin damage, and neuroinflammation in peripheral and autonomic nerves. Structural nerve injury results in impaired nitrergic signalling, reduced nitric oxide (NO) availability, and altered neurovascular coupling, ultimately contributing to erectile dysfunction in diabetes.

## Figures and Tables

**Table 1:** Neuropathic mechanisms of diabetic erectile dysfunction: pathophysiology, assessment tools, and clinical implications.

Neuropathic Domain	Pathophysiological Alteration	Assessment Tools	Clinical Implications for Erectile Dysfunction
**Somatic sensory fibres**	Reduced dorsal penile nerve conduction; loss of Aβ and Aδ fibres	Penile nerve conduction studies; quantitative sensory testing (QST)	Reduced penile sensitivity; impaired afferent input to erectile reflex pathways
**Autonomic** **parasympathetic fibres**	Impaired nitrergic transmission; reduced neuronal nitric oxide synthase (nNOS) activity	Heart rate variability (HRV); pupillometry; autonomic reflex testing	Impaired initiation of erection; reduced response to sexual and sensory stimuli
**Small-fibre neuropathy**	Loss of unmyelinated C fibres and thinly myelinated fibres; altered neurovascular coupling	Corneal confocal microscopy (CCM); quantitative sensory testing	Early erectile dysfunction; poor response to PDE5 inhibitors despite preserved vascular inflow
**Neurovascular coupling**	Disrupted interaction between neural nitric oxide release and endothelial-dependent relaxation	Penile Doppler ultrasound (often within normal ranges); integrated clinical assessment	Erectile dysfunction with preserved penile arterial flow; Doppler findings may underestimate neurogenic impairment
**Neurotrophic support**	Reduced brain-derived neurotrophic factor (BDNF)–TrkB signalling; impaired axonal regeneration	Experimental biomarkers; translational and preclinical models	Limited neural recovery; progressive erectile dysfunction in long-standing diabetes
**Autonomic–urogenital integration**	Concomitant autonomic dysfunction affecting bladder and sexual organs	Lower urinary tract symptom (LUTS) questionnaires; autonomic testing	Frequent coexistence of erectile dysfunction and lower urinary tract symptoms

## Data Availability

No new data were created or analyzed in this study. Data sharing is not applicable to this article.
